# The Heterogeneity of Selective Mutism: A Primer for a More Refined Approach

**DOI:** 10.3389/fpsyg.2021.700745

**Published:** 2021-06-10

**Authors:** Christopher A. Kearney, Melanie Rede

**Affiliations:** Department of Psychology, University of Nevada, Las Vegas, NV, United States

**Keywords:** selective mutism, anxiety disorder, neurodevelopmental disorder, profiles, developmental psychopathology

## Abstract

Selective mutism is a persistent and debilitating psychiatric disorder in which a child fails to speak in situations where speaking is expected. Although listed as an anxiety disorder, the multifaceted and heterogeneous nature of selective mutism indicates that a more accurate conceptualization may be as a neurodevelopmental disorder. This article serves as a primer of historical and clinical presentations, empirical clinical profiles, clinical distinctions, assessment, and treatment related to the complexity of selective mutism. The article includes a brief discussion of selective mutism within a developmental psychopathology perspective with an eye toward reformed efforts for prevention, assessment, and treatment regarding this population.

## Introduction

Selective mutism is a persistent and debilitating mental disorder in which a child fails to speak in settings where speech is expected. Youth with selective mutism often speak well in familiar settings such as home but rarely speak in public settings such as school. Selective mutism interferes with educational or occupational achievement or social communication and must last at least 1 month, excluding the first month of school. The disorder does not usually apply to those with a communication disorder or to those who lack comfort with or knowledge of the primary language spoken in public situations, though the disorder can apply if language skills are adequate. The disorder also, diagnostically, does not occur exclusively among those with autism spectrum or psychotic disorder. Selective mutism is listed as an anxiety disorder in the Diagnostic and Statistical Manual of Mental Disorders and the International Classification of Diseases (DSM-5; American Psychiatric Association, 2013; ICD-11; World Health Organization, 2020). However, the disorder lacks a reference to fear or anxiety in its diagnostic criteria.

Selective mutism is a relatively infrequent disorder with a prevalence rate of about 1–2% (Bergman et al., 2002; Chavira et al., 2004; Sharp et al., 2007). Prevalence may be somewhat higher among immigrant children and those with speech and language delays (Elizur and Perednik, 2003; Manassis et al., 2003). Age of onset is likely in the preschool years though most youth with selective mutism are identified in the early elementary school years (Kristensen, 2000; Cunningham et al., 2004). Selective mutism may be more frequent among males than females in clinical samples but the gender ratio may be more comparable in community samples (Karakaya et al., 2008; Muris and Ollendick, 2015). Selective mutism is a persistent disorder with a variable outcome (Hua and Major, 2016).

Selective mutism is commonly linked to social and other forms of anxiety in many studies, a fact that likely led to its place as an anxiety disorder in contemporary taxonomic systems (Kearney et al., 2019). A growing amount of evidence, however, confirms that youth with selective mutism are quite heterogeneous. Children with selective mutism present with various symptoms that include anxiety, oppositional behaviors, speech and language problems, and features of developmental disorders (Cohan et al., 2008). In addition, the DSM-5 lists many associated features of selective mutism that include not only anxiety-based characteristics but also temperamental (shyness, negativism), social (isolation, withdrawal), and oppositional (temper tantrums) characteristics. The complex clinical picture of those with selective mutism must be fully considered for purposes of individualized assessment and treatment.

The purpose of this article is to briefly summarize clinical and research work, with an emphasis on recent work, that supports a more refined approach for selective mutism based on its heterogeneity and complexity. Key sections in this regard include historical and clinical presentations, empirical clinical profiles, clinical distinctions, assessment, and treatment. A strong link between social and other forms of anxiety with selective mutism is assumed; as such, an emphasis is placed on other features. The article concludes with a discussion of selective mutism within a developmental psychopathology and neurodevelopmental disorder perspective.

## Historical and Clinical Presentations

Selective mutism has been described in various descriptive forms for many decades. Early historical accounts of the condition focused on the voluntary nature of mutism whereby some children would choose to not speak in various settings despite having the capacity to do so (Dow et al., 1995). Other historical accounts reflected a wider range of issues associated with the condition that included aphasia, aphonia, avoidance, fear, inhibition, and trauma, among others (see e.g., Sharkey and McNicholas, 2008). These accounts led to inclusion of elective mutism as a mental disorder in DSM-III and ICD-9 (World Health Organization, 1979; American Psychiatric Association, 1980) that emphasized persistent refusal to speak as well as sensitivity, social withdrawal, and shyness. The phrase “refusal to speak” was later replaced by “failure to speak” (American Psychiatric Association, 1987).

Other descriptive clinical presentations of selective mutism included references to various externalizing behaviors such as argumentativeness, defiance, lying, oppositionality, refusal to attend school, and temper tantrums (Krohn et al., 1992). Others reported irritability, toileting problems, strong-willed behavior, school disobedience, and whining (Steinhausen and Juzi, 1996; Ford et al., 1998; Kumpulainen et al., 1998; Omdal and Galloway, 2007). Negativistic personality traits have been described as well. Youth with selective mutism have been sometimes described as aggressive, controlling, demanding, difficult to please, disobedient, inflexible, manipulative, negative, resistant, stubborn, sulky, and suspicious (Andersson and Thomsen, 1998; Kristensen, 2001; Marakovitz et al., 2011; Vasilyeva, 2013). Externalizing behaviors and negativistic personality traits are not necessarily evident at elevated or clinical levels in this population, however, and may instead represent motivation to avoid or escape anxiety-provoking social situations or obligations as well as attention-seeking behaviors (Yeganeh et al., 2003; Vecchio and Kearney, 2005; Skedgell et al., 2017).

## Empirical Clinical Profiles

Researchers have identified several empirically-based clinical symptom profiles among children with selective mutism that reflect the substantial heterogeneity characteristic of this population. These profiles typically surround themes of anxiety, oppositionality, communication, and other problems. Cohan et al. (2008) asked caregivers of youth aged 5–12 years with selective mutism to evaluate their child's communication delays, expressive and receptive language abilities, functional impairments, internalizing and externalizing symptoms, and social and behavior problems. Latent profile analyses revealed a 3-class solution: anxious-mildly oppositional, anxious-communication delayed, and exclusively anxious. The anxious-mildly oppositional group comprised most of the sample (44.6%) and was characterized by borderline clinical scores for behavior problems and syntax and clinically significant social anxiety scores. Behavior problems were consistent with stubborn or controlling behavior in anxiety-provoking situations. The anxious-communication delayed group also comprised a substantial subset (43.1%) of the sample and was characterized by poor receptive language abilities and syntax as well as clinically significant social anxiety. This group was most impaired and demonstrated greater selective mutism symptom severity and behavior problems than the exclusively anxious group. The exclusively anxious group comprised less (12.3%) of the sample and was characterized by less anxiety and better expressive and receptive language abilities than the anxious-communication delayed group. The study revealed considerable association of oppositionality and speech and language problems with selective mutism.

Mulligan et al. (2015) further identified five subtypes of selective mutism *via* cluster analysis of responses from a clinician-administered measure of symptoms. Global mutism comprised half of the sample and was particularly characterized by less overall and academic impairment and a 2:1 female to male ratio. Low functioning mutism (16.2%) was particularly characterized by academic problems, sensory and executive problems, special education placement, family psychopathology, and an even gender ratio. Sensory/pathology mutism (15.5%) was particularly characterized by bilingualism, motor skill delays, oppositional behavior and lability, sensory integration disorder, separation anxiety problems, an even gender ratio, and greatest impairment. Anxiety/language mutism (10.6%) was particularly characterized by more frequent anxiety and speech and language disorders as well as environmental stress exposure, speech impediments, and a 2:1 female to male ratio. Emotional/behavioral mutism (7.7%) was particularly characterized by executive functioning difficulties, oppositional and labile behavior, and a 10:1 female to male ratio.

Diliberto and Kearney (2016) evaluated parent ratings of internalizing and externalizing behavior problems *via* exploratory and confirmatory factor analysis in a clinical sample of children with selective mutism. Two distinct factors related to anxious and oppositional behaviors were identified. The anxious factor was particularly characterized by a desire to be alone rather than with others, fearfulness/anxiety, nervousness, not eating well, social withdrawal, and sudden changes in mood. The oppositional factor was particularly characterized by argumentativeness, demands for attention, stubbornness, temper tantrums, and whining. Anxious factor scores were linked to other measures of social anxiety and social problems and oppositional factor scores were linked to other measures of aggressive behaviors and oppositional defiant disorder symptoms and inversely to social anxiety disorder symptoms.

Diliberto and Kearney (2018) examined a larger and more diverse sample of children aged 6–10 years identified with selective mutism. Anxiety/distress, oppositionality, and inattention domains were identified *via* initial exploratory and confirmatory factor analysis. Latent class analysis revealed profiles characterized as (1) moderately anxious, oppositional, and inattentive, (2) highly anxious, and moderately oppositional and inattentive, and (3) mildly to moderately anxious, and mildly oppositional and inattentive. The second profile was found to be most impaired and linked to greater emotionality, shyness, and social problems. The third profile was found to be least impaired and linked to better sociability and social competence and activity. The first profile was intermediary to the other profiles with respect to impairment and demonstrated less shyness and social problems than the second profile.

Results from these empirical profiles support the existence of multifaceted anxiety, oppositional, communication, and other symptom patterns among children with selective mutism. These profiles also contain nuanced classes that reveal subtle variations in impairment across different domains. These results have ramifications for classification purposes as well as for refining assessment and case conceptualization strategies in order to identify personalized and perhaps less lengthy treatment. The findings link as well to other recent data on clinical distinctions noted in this population, summarized next.

## Clinical Distinctions

Other research efforts support the notion that selective mutism may be quite distinct from social and other anxiety disorders. Children with selective mutism differ from those with social anxiety in key ways such as behavioral inhibition (Milic et al., 2020), endorsement of speech-demanding situations as more embarrassing (Schwenck et al., 2019), speech-based fears (Vogel et al., 2019), degree of trauma (Mulligan et al., 2015), and anxiety in school-based situations (Poole et al., 2020). Others have noted that children with selective mutism are often rated differently by parents and teachers with respect to behaviors and adaptive skills (Klein et al., 2019).

Reviews also support the notion of heterogeneity among children with selective mutism. Several have noted that oppositional symptoms and/or oppositional defiant disorder may be present in as many as 30% of youth with selective mutism (Alyanak et al., 2013; Kristensen et al., 2019). Meta-analytic results also indicate that children with selective mutism are diagnosed with an anxiety disorder in only 80% of cases (69% for social phobia) (Driessen et al., 2020). In many of these cases, unrelated diagnoses of specific phobia are common. Rozenek et al. (2020) also concluded in their review that selective mutism was part of a heterogenic group of disorders with a multifaceted, overlapping, and complex etiology.

Others have noted that selective mutism relates as well to various neurodevelopmental and communication disorders. Steffenburg et al. (2018) found that nearly two-thirds (63%) of their sample of children with selective mutism had a comorbid autism spectrum disorder. In addition, children with selective mutism and autism often demonstrated speech delays and intellectual disabilities. Others have noted similar features of autism among children with selective mutism (Cengher et al., 2020; Suzuki et al., 2020). Communication difficulties are common in this population as well and may be especially prevalent among those with sensory and anxiety problems (Mulligan et al., 2015). Speech and language problems among children with selective mutism often include difficulties with detailed narratives, discrimination of speech sounds, grammar, phonological awareness, and receptive language (Klein et al., 2013). Collectively these findings point to the possibility that selective mutism is a neurodevelopmental disorder involving speech and language that may be impacted by changes in auditory efferent feedback pathways, vagal responses, genetics, or other physiological factors (Heilman et al., 2012; Young et al., 2012; Muchnik et al., 2013; Henkin and Bar-Haim, 2015).

## Assessment

The heterogeneity of selective mutism is also mirrored by the many recommended assessment procedures and targets for the disorder. A particular focus is made on evaluating the parameters and function of a child's failure or refusal to speak in addition to social and other forms of anxiety, oppositional problems, communication deficits, and/or intellectual disabilities (Mayworm et al., 2015). Assessment for this population thus typically includes audio/video recordings, behavioral observations, formal testing, interviews, and questionnaires (e.g., Selective Mutism Questionnaire; Bergman et al., 2008) for children, parents, and teachers (Shriver et al., 2011). Specific information gathered often includes compensatory behaviors for non-speaking, contextual factors that impact non-speaking, interference with academic and social functioning, operant factors that maintain mutism, and responses from key others to a child's mutism (Kearney, 2010). Operant factors can include inefficient or underdeveloped speaking skills as well as motivation to decrease anxiety, increase social or sensory (physical) feedback from others, and/or avoid aversive directives from others (Skedgell et al., 2017).

With respect to speech, specific information gathered can include various settings in which a child fails to speak as well as the range of speaking behavior (e.g., low volume speech mouthing, whispering) in each setting (Kearney et al., 2019). Additional critical information includes to whom a child will speak in different situations, communication and articulation problems, and language differences (Oerbeck et al., 2018). School-based assessments are important as well and can include a child's interactions with peers and teachers (including threats from others), avoided situations, and performance on different academic tasks (Hua and Major, 2016). Formal assessment of intellectual/achievement and speech/language abilities are often conducted at school as well and can focus on performance on non-verbal tasks, receptive language, and written narratives as well as academic records and teacher interviews (Martinez et al., 2015).

## Treatment

The heterogeneity of selective mutism is also represented by the many multimodal treatment packages that researchers and others have used to best account for the different characteristics of this population (Østergaard, 2018). Common intervention elements thus include exposure-based practices, family therapy, group therapy, parent-based contingency management, self-modeling, shaping and prompting, social skills and language-based training, and stimulus fading as well as pharmacotherapy (Manassis et al., 2016; Klein et al., 2017). Use of digital technology is sometimes a part of these interventions as well (Bunnell et al., 2018). These elements are designed to enhance audibility and frequency of speech and to ameliorate competing behaviors and dynamics that interfere with appropriate speech (Zakszeski and DuPaul, 2017).

Recent multimodal treatment efforts have broadened these efforts toward intensive and group-oriented interventions. Lorenzo et al. (2020) used a blended approach of parent-child interaction therapy, live parent coaching and child directed interaction, and cognitive-behavioral techniques such as stimulus fading in a multi-day, all-day group format in addition to videoconferencing and school outreach to address young children with selective mutism. Cornacchio et al. (2019) utilized a similar approach to find that intensive group therapy produced larger remissions of selective mutism, reductions in social anxiety, and long-term improvements in functional outcomes compared to waitlist control. Skedgell et al. (2017) utilized personalized individual and group therapy with exposure-based and contingency management procedures to effect change in young children with selective mutism. Indeed, the inclusion of multiple parties is now considered an essential aspect of treatment for selective mutism (Catchpole et al., 2019).

## Developmental Psychopathology

Several key tenets of developmental psychopathology potentially apply well to selective mutism, including biological foundations, multiple pathways (multifinality), and cascading effects. In addition, the heterogeneous, complex, and multifaceted nature of selective mutism may indicate that the disorder is better classified as a neurodevelopmental disorder than as an anxiety disorder. First, as mentioned, selective mutism may be a disorder with abnormalities in central nervous system development and epigenetic foundations (Henkin and Bar-Haim, 2015). Atypical neurodevelopment of speech in selective mutism is reflected in the many communication disorders evident in this population as well as the relative lack of findings regarding consistent environmental causes for the disorder. Genetic and neuroimaging work support this idea as well (Peñagarikano and Geschwind, 2012; Eugene and Masiak, 2016).

Features of neurodevelopmental disorders involve core characteristics as well as surrounding clinical profiles that can include developmental, cognitive, behavioral, and emotional characteristics (Thapar et al., 2017). For selective mutism, the core characteristic of non-speaking is often surrounded by developmental (e.g., speech delays), cognitive (e.g., social fears), behavioral (e.g., oppositional), and emotional (e.g., temperamental) characteristics. In addition, neurodevelopmental disorders, as with selective mutism, are marked by shared risk factors; manifested by heterogeneity in symptoms, outcome, and treatment response without clear boundaries across the disorders; impacted heavily by social context such as demands and resources; and subject to developmental change and various avenues of progression across the lifespan (Andrews et al., 2009; Thapar et al., 2017).

Second, selective mutism may be particularly amenable to the concept of multifinality or the fact that multiple pathways involving collections of different risk factors can lead to various profiles of the disorder (Kearney et al., 2019). Children with selective mutism may begin with an initial neurodevelopmental etiology that later progresses toward different clinical profile pathways. One key pathway may be largely based on anxiety profiles, with risk factors such as early genetic and behavioral inhibition predispositions and later environmental factors such as interpersonal distress and social avoidance. Another key pathway may be largely based on oppositional profiles, with risk factors such as early temperamental differences as well as family dynamics, parent/teacher/peer responses, and operant variables that contribute to coercive interactions, dependence on others for communication, and non-compliance. Still another key pathway may be largely based on communication profiles, with risk factors such as early deficits in in speech, language, or learning as well as later problems in social interactions and academic performance.

Third, selective mutism may represent a key inflection point or marker in development that impedes a child's ability to advance well in other important domains of functioning, thus exacerbating the disorder. Selective mutism could thus be conceptualized from a developmental cascade approach whereby problems in one domain of functioning spreads to deficits in other domains of functioning (Panayiotou and Humphrey, 2018). Failure to speak during the toddler and preschool period could impede the development of executive functioning skills and social competencies that lead to early, school-based difficulties in academic performance and social interactions (Vogan et al., 2018). Deficits in academic and social competencies are closely linked to later internalizing (e.g., anxiety) and externalizing (e.g., oppositionality) behavior problems (Hu et al., 2015). A cascading effect of these multiple, interacting problems can then lead to substantial broader problems such as extensive social withdrawal, specialized academic placement, and school disengagement that can help explain the persistence of selective mutism (Mulligan et al., 2015).

## Conclusion

Emerging, sprawling, and interdisciplinary work on selective mutism reveal the disorder to be heterogeneous, complex, and multifaceted. In many ways, evolving conceptualizations of selective mutism reflect longstanding historical views of this population. The notion of selective mutism as a neurodevelopmental disorder carries many implications for prevention, assessment, and intervention. Examples regarding prevention include public health initiatives, efforts to improve maternal health, early assessment and speech and language training, and expanded preschool opportunities. Clinical and school-based professionals should collaborate closely to screen preschoolers for possible neurodevelopmental problems that could include selective mutism and its pathways.

Examples regarding assessment include efforts to expand evaluative efforts beyond simple speaking behaviors and to broaden case conceptualization to include biological and other contributing variables. Clinical and school-based professionals should collaborate closely in cases of selective mutism to comprehensively evaluate likely comorbid psychiatric and developmental problems such as anxiety/oppositionality with communication problems and autism spectrum disorder/intellectual disability. Examples regarding intervention include efforts to recognize the whole child with selective mutism, whether it be in clinical treatment or in school-based accommodation plans, to nurture multiple areas of a child's development. Clinical and school-based professionals should collaborate closely in the development of these plans to chart appropriate goals and evaluate progress. This article was designed to offer an initial platform for expanding the conceptualization of selective mutism to help advance these efforts.

## Author Contributions

CK and MR contributed to the writing and editing of the manuscript.

## Conflict of Interest

The authors declare that the research was conducted in the absence of any commercial or financial relationships that could be construed as a potential conflict of interest.
